# Rice *RBH1* Encoding A Pectate Lyase is Critical for Apical Panicle Development

**DOI:** 10.3390/plants10020271

**Published:** 2021-01-30

**Authors:** Dong He, Rui Liang, Tuan Long, Ying Yang, Changyin Wu

**Affiliations:** 1 National Key Laboratory of Crop Genetic Improvement, National Center of Plant Gene Research (Wuhan), Huazhong Agricultural University, Wuhan 430070, China; sxliangrui@gmail.com (R.L.); hnlongtuan@gmail.com (T.L.); yangyinghb@gmail.com (Y.Y.); 2 College of Tropical Crops, Hainan University, Haikou 570100, China; 3 The Center of Plant Science Innovation, University of Nebraska–Lincoln, Lincoln, NE 68588, USA

**Keywords:** rice, *RBH1*, panicle development, pectate lyase, ROS

## Abstract

Panicle morphology is one of the main determinants of the rice yield. Panicle abortion, a typical panicle morphological defect results in yield reduction due to defective spikelet development. To further elucidate the molecular mechanism of panicle abortion in rice, a *rice panicle bald head 1* (*rbh1*) mutant with transfer DNA (T-DNA) insertion showing severely aborted apical spikelets during panicle development was identified and characterized. The *rbh1-1* mutant showed obviously altered cell morphology and structure in the degenerated spikelet. Molecular genetic studies revealed that *RBH1* encodes a pectate lyase protein. Pectate lyase-specific activity of Rice panicle Bald Head 1 (RBH1) protein assay using polygalacturonic acid (PGA) as substrates illustrated that the enzyme retained a significant capacity to degrade PGA. In addition, immunohistochemical analysis showed that the degradation of pectin is inhibited in the *rbh1-1* mutant. Further analysis revealed that a significant increase in reactive oxygen species (ROS) level was found in degenerated *rbh1-1* spikelets. Taken together, our findings suggest that *RBH1* is required for the formation of panicle and for preventing panicle abortion.

## 1. Introduction

In rice, the mechanisms of panicle development have been studied preliminary by research on a number of genes. *FRIZZLE PANICLE* (*FZP*), as the main negative regulator of *ABERRANT PANICLE ORGANIZATION 2* (*APO2*), regulates spikelet formation, and identifies the fate of floral organs by regulating the expression of MCM1, AG, DEFA, and SRF (*OsMADS*)-box genes [1,2,3,4]. The rice *MONOCULM1* (*MOC1*), *LAX PANICLE1* (*LAX1*) and *LAX PANICLE2* (*LAX2*) genes control the initiation and maintenance of the axillary meristem during the vegetative phase and rachis-branch meristem during the reproductive phase. The mutation of *MOC1*, *LAX1,* and *LAX2* leads to defective panicle development and fewer tillers [5,6,7,8]. All of the above genes are involved in the initiation of the branch meristem and the differentiation of the spikelet primordia, but the research focused on the regulation of branch elongation and floret formation is deficient.

The panicle abortion that is common in the crop breeding occurs mainly during the branch elongation and floret formation. Recently, researchers identified several genes related to panicle abortion and elucidated basic biochemical functions of these pivotal genes. *TUTOU1* (*TUT1*) is a functional suppressor of cAMP receptor/Wiskott–Aldrich syndrome protein family verprolin-homologous (SCAR/WAVE) and activates actin nucleation and polymerization. The *tut1* mutant shows degenerating spikelet in the apical parts of all primary and secondary branches [9]. *Aluminum-activated Malate Transporter* 7 (*OsALMT7*) mediating malate transport is critical for the maintaining apical spikelet and grain yield. The loss of function of *OsALMT7* results in a pleiotropic phenotype, including panicle apical abortion and short panicle length [10]. Physiological and biochemical experiments indicated that the loss of function of *SQUAMOSA PROMOTER-BINDING PROTEIN-LIKE 6* (*SPL6*) gene could bring out the up-regulation of *Inositol-Requiring Enzyme 1* (*IRE1*), eventually leading to cell death in the rice panicle. The *spl6* mutant has pale glumes and serious apical spikelet abortion [11,12,13]. The disruption of *Calcineurin B-Like Protein-Interacting Protein Kinase 31* (*OsCIPK31*) would result in the cell death during panicle development. *OsCIPK31* and mitogen-activated protein kinase (MAPK) pathway may interact in a response to stress by increasing reactive oxygen species (ROS) accumulation. The *oscipk31* mutant displays reduced spikelet number per panicle and brown lesions on glumes [14].

Together with many complex natural plant polymers, pectin is the major component of plant cell wall [15,16,17]. Considerable research has indicated that pectin participates in numerous biological processes, including cell wall deposition and cell expansion [18], cell intercellular adhesion [19], cell wall swelling and softening during fruit ripening [20], cell separation during fruit abscission, pod dehiscence, and root shoot cell differentiation [21,22]. Homogalacturonan (HG), as important pectin substance, is usually highly methyl esterified. Pectin methylesterases (PMEs) can effectively reduce the level of methyl esterification in HG [23]. Pectate lyases-like (PLs) contain pectate lyases (endo-PLs and exo-PLs) and pectin lyases (endo-PNLs) [24,25,26]. PLs specially degrade non-methylesterifed or poorly methylesterifed HG. Ca^2+^ and pH 8.5 are necessary for the activity of PLs [27,28]. Previous study pointed out that PLs gene was originally found in *Erwinia carotovora* [29]. However, most evidence indicated that PLs sequences are abundant in plant genomes. Currently, the genome sequences homology analysis predicts there are 26 PLs genes in Arabidopsis and 14 in rice [30,31]. In addition, these PLs genes are widespread in various plant species, including tomato, tobacco, alfalfa, and Chinese cabbage [32,33,34,35].

Some genes encoding pectin lyase are identified based on molecular biology and genetics. *LATE ANTHER TOMATO 56* (*LAT 56*) and *LATE ANTHER TOMATO 59* (*LAT 59*), the first identified PLs genes in tomato, have high sequence similarity with the *Erwinia carotovora* PLs gene and are expressed strongly in mature flower organs [36]. In Arabidopsis, *Powdery Mildew Resistance 6* (*PMR6*) encodes a pectate lyase. The mutation in *PMR6* leads to alteration of the plant cell wall composition and effectively improves the resistance to powdery mildew [37]. In addition, *Lotus japonicus nodulation pectate lyase* (*LjNPL*) encodes pectate lyase and is induced by rhizobial nodulation factors. The function of *LjNPL* in plant cell wall degradation is essential for nodule infection by rhizobia [38]. The *yellow margin* mutant has small and round leaves and shortened plant height. Correspondingly, the *Yellow Margin* gene encodes a pectate lyase-like protein and regulates cell expansion in potato [39]. 

In addition to the role of pectate lyase associated with plant pathogen infection, we have a preliminary understanding of the importance of pectin lyase in plant development. However, the function of pectin lyase in plant growth, especially in panicle development in rice is poorly understood. Here, we report that the *Rice panicle Bald Head 1 (RBH1)* gene encoding pectate lyase is responsible for apical spikelet maintenance and panicle growth in rice. Two alleles of *rbh1* mutant show defective panicle, including whitish, twisty spikelet and degenerated floral organs. The RBH1 degrades pectic substances, and the mutation of *RBH1* enhances ROS accumulation. Our results demonstrate that *RBH1* is essential for preventing panicle abortion and maintaining panicle development.

## 2. Results

### 2.1. Identification of rbh1-1 Mutant

**Figure 1:**
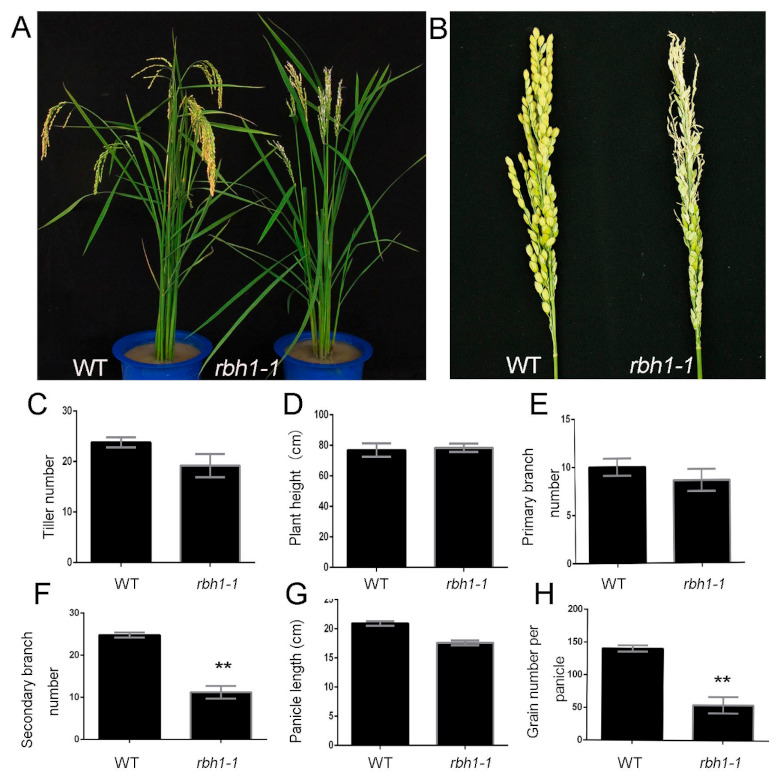
Morphology of *rbh1-1* mutant and wilt type (WT) at the heading stage. (**A**) Phenotype comparison of WT (left) and *rbh1-1* (right). (**B**) Phenotype comparison of WT panicle (left) and *rbh1-1* representative panicle (right). (**C–H**) Agronomic trait analysis of tiller number (**C**), plant height (**D**), primary branch number (**E**), secondary branch number (**F**), panicle length (**G**), and grain number per panicle (**H**) between WT and *rbh1-1*. Data are presented as means ± SE (n = 5). ** *p* < 0.01 (Student’s *t*-test).

To understand the molecular and genetic mechanisms of the panicle developmental regulation, we screened mutant with apical spikelet defect in the experimental field. One mutant *rbh1-1* with abnormal panicle phenotype was identified. The mutant plant differed from the wild type (WT) plant by having an obvious apical panicle defect. The formation of terminal spikelets in mutant plant were severely inhibited and replaced by twisty, whitish spikelets (Figure 1A,B). The agronomic traits data showed no obvious difference between the mutant and the WT during the reproductive growth stage in number of tillers (Figure 1C), plant height (Figure 1D), number of primary branches (Figure 1E), and panicle length (Figure 1G). Consistent with the observed panicle phenotype, the number of secondary branches (Figure 1F) and grains per panicle (Figure 1H) were significantly reduced in the mutant. These results suggest that *RBH1* is necessary for the panicle development.

### 2.2. Spikelet Mutation Phenotype of rbh1-1 Mutant

**Figure 2:**
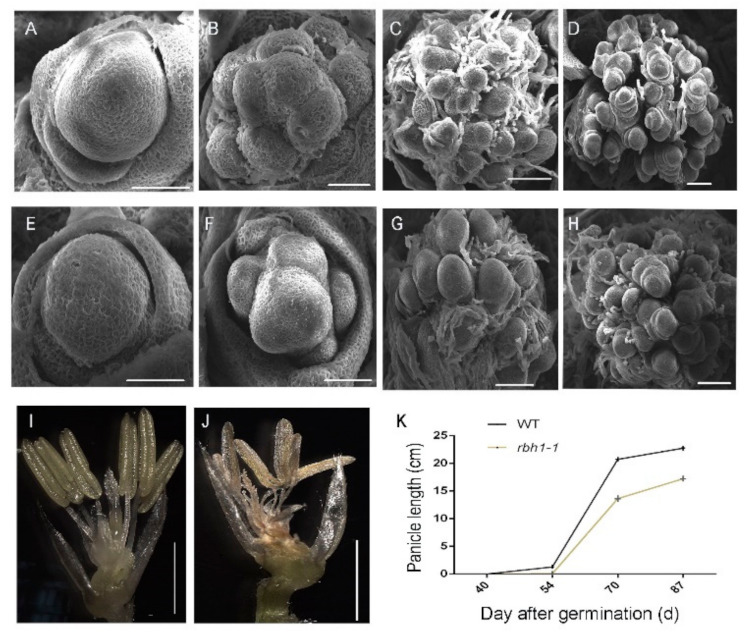
Observation of the abnormal panicle development in *rbh1-1* mutant. (**A–H**) Scanning electron microscope (SEM) images showing the development of young panicles in the WT (**A–D**) and *rbh1-1* (**E–H**): the formation of SAM (**A**,**E**), the formation of primary branch primordia (**B**,**F**), the formation of secondary branch primordia (**C**,**G**), and the formation of floret primordia (**D**,**H**). Bar = 50 μm (**A**,**B**,**E**,**F**), bar = 100 μm (**C**,**D**,**G**,**H**). (**I**,**J**) Structure of the representative spikelet in WT (**I**) and *rbh1-1* (**J**) at the heading stage. Bar = 2 mm. (**K**) The panicle length during panicle growth in WT and *rbh1-1*.

To clarify the panicle developmental defect in the *rbh1-1* mutant, we compared the process of rice panicle formation between WT and *rbh1-1* mutant during early panicle development. Scanning electron microscopic observations showed there was no obvious morphological difference in the shoot apical meristem (SAM) between the WT and *rbh1-1* mutant (Figure 2A,E). During the subsequent primary branch primordia development, the *rbh1-1* mutant and WT showed similar morphology (Figure 2B,F). The mutant showed normal morphological structure during the secondary branch primordia development (Figure 2C,G). During the formation of floret primordia, there was no obvious defect in the mutant (Figure 2D,H). Using a stereomicroscope, we found that the flower organs in *rbh1-1* were distorted and the color was darker (Figure 2I,J). To further elucidate the defect of apical spikelet in mutant, we compared the growth rate of WT and mutant panicles. We found that the mutant showed reduced panicle growth rate (Figure 2K); the panicle growth data of *rbh1-1* mutant and WT were shown in Supplementary Table S1. The above observations indicated the mutant phenotype of *rbh1-1* was mainly due to suppressed panicle development rather than the early termination of apical spikelet primordia development. Our results indicate that *RBH1* might not function in the early stage of panicle development, but participated in panicle development during the stage of panicle elongation.

### 2.3. Gene Cloning and Genetic Complementary Test

**Figure 3:**
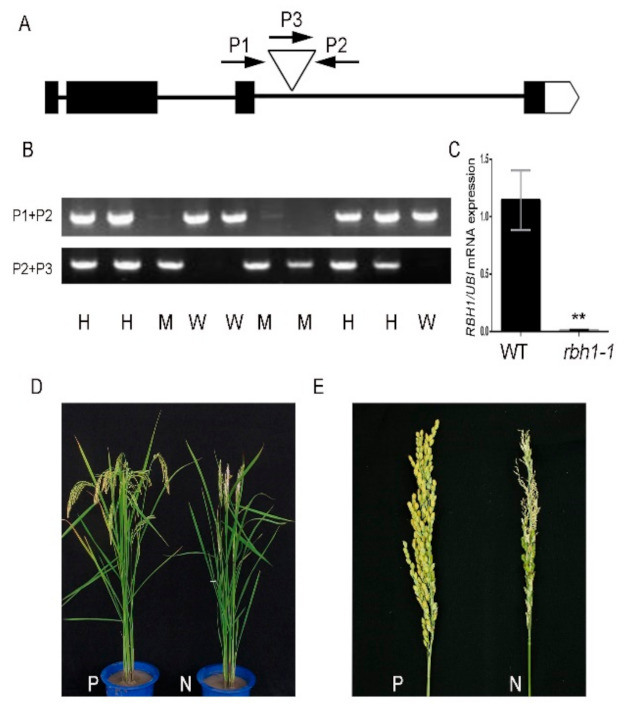
Identification of *RBH1*. (**A**) The structure of *RBH1* and Transfer DNA (T-DNA) insertion sites. Black lines represent the intron, the black boxes represent the exon, the white arrow represents the 3’UTR region, and the white triangle represents the T-DNA insertion site. P1 and P2 are genomic primers on both sides of T-DNA insertion site, P3 is a T-DNA boundary primer. (**B**) PCR genotyping in the *rbh1-1* segregant. All plants homozygous for T-DNA insertion showed the positive band with P2 + P3 primers and those with the negative band with P1 + P2 primers have mutant phenotype (M). All WT plants (W) showed the positive band with P1 + P2 primers and the negative one with P2 + P3 primers. Plants heterozygous for T-DNA insertion showed both positive bands have normal phenotype (H). (**C**) qRT-PCR analysis of *RBH1* expression in WT and *rbh1-1* panicle (1—5 mm). The rice *ubiquitin* (*UBI*) gene was used for normalization. Data are presented as means ± SE (n = 3). ** *p* < 0.01 (Student’s *t*-test). (**D**) Phenotypes of the transgenic positive (P) and transgenic negative (N) plants at the heading stage. (**E**) Mature panicles of the transgenic positive (P) and transgenic negative (N) plants.

To confirm that the defective apical panicle phenotype was due to the T-DNA insertion, the genomic sequence flanking the insertion site was amplified by thermal asymmetric interlaced-polymerase chain reaction (PCR) [40]. This result revealed that the T-DNA tag was located in the third intron of Loc_Os10g31910 (*rbh1-1* approximate insertion site: 3340 bp) (Figure 3A). Loc_Os10g31910 consists of four exons and three introns, and encodes a putative pectate lyase (Figure 3A). To verify whether the defective apical panicle phenotype was caused by the T-DNA insertion in *RBH1*, a pair of gene-specific primers P1, P2 and a T-DNA sequence-specific border primer P3 were used to detect the genotype of the heterozygous population. All the plants with homozygous T-DNA insertion showed the phenotype of the apical spikelet defect, and the other plants without T-DNA insertion or with heterozygous T-DNA insertion showed normal panicle morphology (Figure 3B). Then we examined the expression of *RBH1* in the WT and *rbh1-1* mutant. Quantitative reverse transcription PCR (qRT-PCR) result showed that the *RBH1* transcript was significantly decreased in the *rbh1-1* mutant compared to WT (Figure 3C). These results suggest that the mutation of *RBH1* results in abnormalities in the apical spikelets. 

In addition, another T-DNA insertion line designated as *rbh1-2*, in which the T-DNA insertion site was located in the 3^rd^ intron of *RBH1* (approximate insertion site: 3693 bp) (Supplementary Figure S1I) showed the same apical spikelet defect phenotype as that in the *rbh1-1* mutant (Supplementary Figure S1A,B). The agronomic traits data showed no obvious difference in plant height (Figure S1D) and number of primary branches (Supplementary Figure S1E) between the WT and *rbh1-2* mutant, whereas the number of tillers (Supplementary Figure S1C), secondary branches (Figure S1F), panicle length (Figure S1G), and grains per panicle (Supplementary Figure S1H) were significantly reduced in the *rbh1-2* mutant. Finally, the T-DNA insertion in *RBH1* co-segregated with the mutant phenotype in the *rbh1-2* mutant, as in the case of *rbh1-1* (Supplementary Figure S1J).

To further confirm that apical spikelet abnormalities were caused by mutation in *RBH1*, a fragment of genomic DNA containing a complete *RBH1* coding region and a 2877-bp upstream DNA fragment was introduced into the *rbh1-1* mutant background. Under the natural growth conditions, the T_1_ lines that were self-crossed by the T_0_ transgenic plants displayed the phenotype segregation (Supplementary Table S1), and all the transgenic individuals reverted to a normal panicle phenotype (Figure 3D,E). Therefore, we propose that the apical panicle defect of *rbh1-1* is caused by the mutation of *RBH1*.

### 2.4. Expression Analysis of RBH1 and Sequence Analysis of RBH1

**Figure 4:**
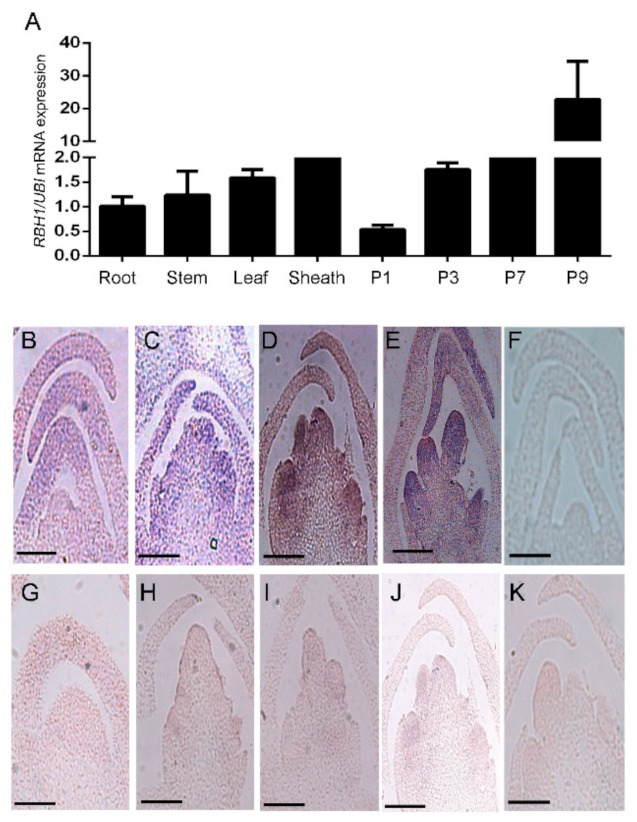
Expression pattern of *RBH1*. (**A**) Expression profiles of *RBH1* in the root, stem, leaf, sheath, and panicles. P1, P3, P7, and P9 represent tissues of rice panicles of 1, 3, 7, and 9 cm long, respectively, before heading. The rice *UBI* gene was used for normalization. Data are presented as means ± SE (n = 3). (**B–E**) In situ hybridization with a *RBH1* antisense probe on a longitudinal section of a shoot during development stages in WT. Bar = 100 μm. (**G–J**) In situ hybridization with a *RBH1* antisense probe on a longitudinal section of a shoot during development stages in *rbh1-1*. Bar = 100 μm. (**F**,**K**) In situ hybridization with a *RBH1* sense probe (negative control) on a longitudinal section of the vegetative shoot in WT and mutant. Bar = 100 μm.

To investigate the expression pattern of *RBH1* in rice, the qRT-PCR was carried out to examine the expression of *RBH1*. The results showed that *RBH1* was detected in all the examined tissues, especially in the panicle development stage, and the expression level of *RBH1* was significantly enhanced during panicle elongation (Figure 4A). To further analyze the spatial expression pattern of *RBH1*, the *in situ* hybridization was designed to detect the expression of *RBH1* in young panicle. The result showed that the *RBH1* transcript was detected during the whole process of early panicle development (Figure 4). At the SAM stage, the expression of *RBH1* was at a low level (Figure 4B), but the *RBH1* expression increased gradually during the primary branch (Figure 4C) and secondary branch development (Figure 4D). The *RBH1* mRNA accumulation was maximized during the formation of floret primordium (Figure 4E). These results illustrate that the *RBH1* is a constitutively expressed gene and *RBH1* is essential in panicle development, especially in the process of panicle elongation.

In rice, *RBH1* encodes a typical pectate lyase containing 491 amino acid residues (Supplementary Figure S2). Sequence analysis revealed that RBH1 protein shared the conserved Amb_all domain with other pectate lyase proteins. In addition, the RBH1 protein, PMR6 (pectate lyase required for powdery mildew susceptibility in Arabidopsis) [37] and LjNPL (legume pectate lyase required for root infection by rhizobia) [38] did not contain the Pec_lyase_N domain (Supplementary Figure S2). The high degree of homology in the amino acid sequences among RBH1, PMR6, and LjNPL suggests that these proteins may have evolutionarily conserved biochemical function.

### 2.5. The Function of RBH1 Protein

**Figure 5:**
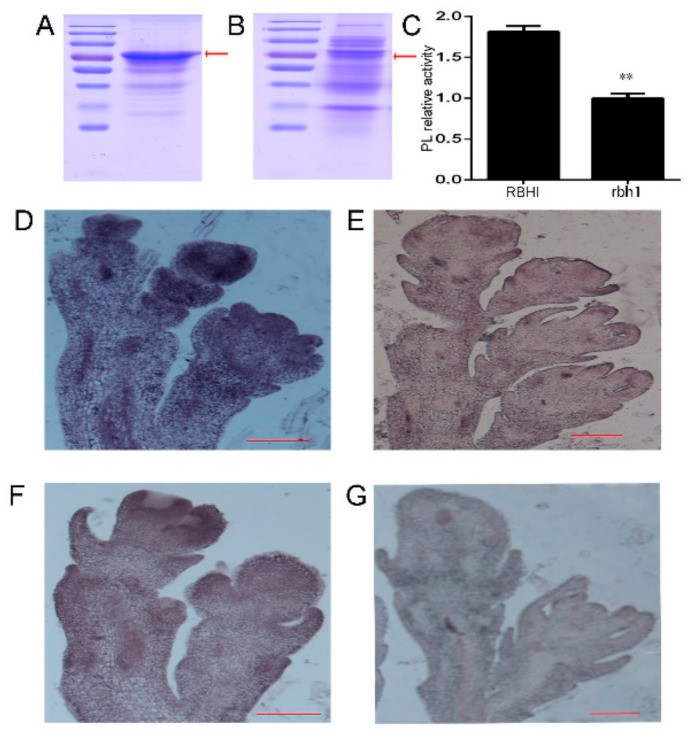
Biochemical characterization of RBH1. (**A**,**B**) Sodium Dodecyl Sulfate polyacrylamide gel electrophoresis (SDS-PAGE) of the WT (**A**) and rbh1 (**B**) Maltose Binding Protein (MBP)-tagged pectate lyases. (**C**) Pectate lyase-specific activities of the WT and rbh1 proteins assayed using polygalacturonic acid (PGA) as the substrate. Data are presented as means ± SE (n = 3). ** *p* < 0.01 (Student’s *t*-test). (**D–G**) Immunolocalization of HG in the *rbh1-1* (**D**,**F**) and WT (**E**,**G**) plants using JIM5 antibodies in *rbh1-1* (**D**) and WT (**E**), and LM18 antibodies in *rbh1-1* (**F**) and WT plant (**G**). Scale bar = 100 μm.

In order to determine the biochemical function of *RBH1*, it is necessary to assess the possible pectate lyase activity of RBH1 in vitro. Bioinformatics predicted that RBH1 protein may have transmembrane domains (https://services.healthtech.dtu.dk/service.php?TMHMM-2.0). We were not successful in inducing RBH1 full-length protein using prokaryotic expression system. Then, we constructed the RBH1 truncated sequences to represent RBH1 (51-465aa) and *rbh1* (51-422aa) proteins, then purified them using the purification system of N-terminal Maltose Binding Protein (MBP) tagging. Sodium Dodecyl Sulfate polyacrylamide gel electrophoresis (SDS-PAGE) suggested that WT and mutant proteins were approximately 80 kDa (Figure 5A,B). Sequence analysis revealed that RBH1 and LjNPL shared a high degree of homology (Supplementary Figure S2), and previous studies have reported that *LjNPL*-encoding pectate lyase degraded the substrate polygalacturonic acid (PGA) in vitro [38]. We verified the enzymatic activity of RBH1 using the purified wild type and mutant proteins. The purified wild type RBH1 protein retained a significantly higher capacity to degrade polygalacturonic acid than the rbh1 protein (Figure 5C).

To determine whether RBH1 had the capacity to degrade pectin in vivo, the immunohistochemical assay was designed to detect galacturonic acid in WT and the mutant. JIM5 and LM18 are commercial antibodies for detection of pectin in plants. These antibodies were used to recognize partially demethylesterified and non-methylesterifed HG [41,42]. On the whole, the signal intensity was more prominently detected in *rbh1-1* (Figure 5D, F) compared to WT (Figure 5E,G). The intensity of the JIM5 hybridization signal peaked in the apical area of the young spikelet where the flower primordium developed (Figure 5D). The signal distribution pattern of the LM18 antibody was basically consistent with that of the JIM5, showing the intense hybridization signal in the area of the floret primordia formation (Figure 5F). The above results indicate that the degradation of pectin is inhibited in the *rbh1-1* mutant, and therefore it has a high concentration of pectin in the panicle tissue. Moreover, the increased accumulation of pectin in the floret primordia of mutant panicle also suggests that the normal degradation of pectin during early panicle development is necessary for the formation of floret primordia. 

### 2.6. Subcellular Structure of rbh1-1 Mutant

In order to determine whether the increased accumulation of pectin in the *rbh1-1* mutant resulted in change of *rbh1-1* spikelet cell morphology and structure, we observed the morphology of *rbh1-1* and WT spikelets by transmission electron microscopy. We observed intact cell structure in both WT and *rbh1-1* spikelets (Supplementary Figure S3A,B). The cells of the WT spikelet were uniform in size and orderly arranged (Figure S3A). In comparison, the *rbh1-1* spikelet cells were disorderly arranged and irregular in shape (Supplementary Figure S3B). Therefore, we propose that the mutation of *RBH1-1* gene leads to a significant change in the structure of spikelet cells.

### 2.7. The RBH1 Mutation Enhanced the ROS Accumulation

Previous reports have shown that mechanical stress, a kind of abiotic stress, may disturb the dynamic balance of ROS production and degradation [43]. In addition, mechanical stimulation such as cell expansion could trigger an increase in the cytosolic Ca^2+^ concentration and ultimately lead to activation of ROS production [44,45]. Our results indicated that the cell morphology and structure of *rbh1-1* mutant were changed (Supplementary Figure S3). Whether this change in plant internal environment would alter ROS concentration warrants further work.

**Figure 6:**
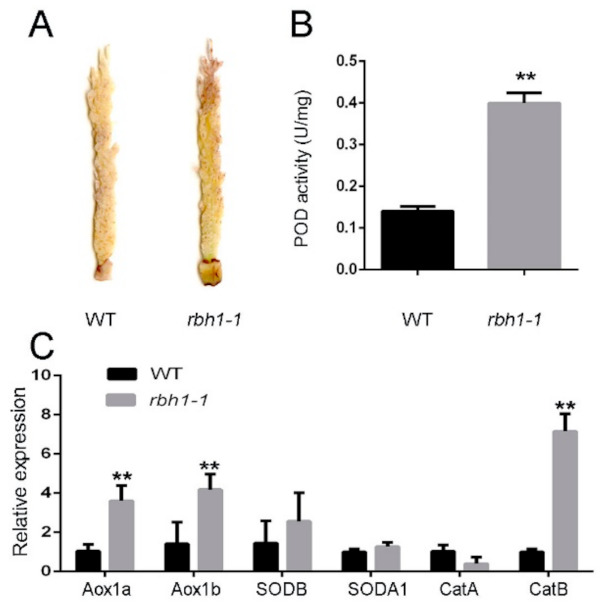
ROS accumulation in WT and *rbh1-1*. (**A**) 3,3′-diaminobenzidine (DAB) staining of the WT and *rbh1-1* panicles. (**B**) Peroxidase (POD) activity in the WT and *rbh1-1* panicles. (**C**) Relative expression of genes related to reactive oxygen species (ROS) scavenging in the WT and *rbh1-1* panicles. Data are presented as means ± SE (n = 3). The rice *UBI* gene was used for normalization. ** *p* < 0.01 (Student’s *t*-test).

In order to confirm the change in ROS concentration in the *rbh1-1* mutant, we performed the 3,3′-diaminobenzidine (DAB) staining test to detect H_2_O_2_ accumulation. The *rbh1-1* plants showed more extensive staining than WT (Figure 6A). Peroxidase (POD) as one of the antioxidative enzymes that remove excessive ROS can effectively detoxify H_2_O_2_ to H_2_O [46]. As expected, our results suggested that the activity of POD in the *rbh1-1* panicle was greatly increased compared with the wild type panicle (Figure 6B). It was already reported that the alternative oxidases (AOX) genes, superoxide dismutase (SOD) genes and catalase (CAT) genes functioned coordinately in the ROS-scavenging pathways in response to the aberrant abundance of intercellular ROS [47,48,49,50,51]. The qRT-PCR was performed to measure transcript levels of these ROS-scavenging genes. *AOX1a*, *AOX1b*, and *Catb* were significantly increased in the *rbh1-1* panicle (Figure 6C). These results indicate that the mutation of *RBH1* gene results in significantly increased ROS level in the defective *rbh1-1* spikelets.

## 3. Discussion

### 3.1. Mutation in RBH1 Resulted in Obvious Apical Panicle Defect

Panicle development is a complex biological process regulated by many genes. Earlier research has showed that some key genes such as *LAX1* and *LAX2* are mainly involved in the initiation/maintenance of rice axillary meristem. The corresponding mutant phenotypes of these genes show fewer rachis-branches and suppressed lateral spikelets [5,8]. In the study presented here, the loss of function of *RBH1* gene resulted in the apical spikelet defect (Figure 1) and *RBH1* positively regulated the formation of apical spikelet and panicle growth rate (Figure 2). Furthermore, the expression of *LAX1* and *LAX2* was upregulated significantly in the initial region of axillary meristem [5,8]. In our study, the expression of *RBH1* was detected throughout the development of panicle, and increased significantly in mature panicle (Figure 4A). It is considered that *RBH1* differed from *LAX1* and *LAX2* regulates the panicle development in an independent pathway. Our research suggests that the specific function of *RBH1* is indispensable to maintain the natural development of apical panicle. Moreover, the previous study suggested that panicle abortion caused mainly by unfavorable conditions such as extreme temperature or drought stress is unstable and susceptible to abiotic stresses [52]. The rice panicle abortion resulting in significant reduction in the number of effective spikelets and ultimately leading to depressed yield has not been clarified completely. The identification and function analysis of *RBH1* gene provides new information for preventing apical panicle abortion in rice breeding.

### 3.2. RBH1 Encodes a Pectate Lyase Involved in Pectin Degradation 

It is well known that PLs genes play a crucial role in a series of growth and development processes, for example pollen tube emergence [33,34], tracheary element maturation [53,54], and fruit ripening [55,56], and are also important in the resistance to plant pathogens [37] and response to plant hormones and environmental stresses [30,53]. In particular, the PLs gene *Oryza sativa premature senescence 1* (*OsPSE1*) identified by mutant analysis is involved in leaf senescence [31]. The knockdown mutation of two PLs genes *Oryza sativa* *Pectate lyase-like 3* (*OsPLL3*) and *Oryza sativa* *Pectate lyase-like 4* (*OsPLL4*) results in disrupted pollen development and gives rise to partial male sterility [57]. In our study, the formation of terminal spikelets in the *rbh1-1* mutant was severely inhibited and replaced by twisty, whitish spikelets (Figure 1A,B). Additionally, a high degree of homology in amino acid sequence between RBH1, PMR6 [37] and LjNPL [38] implied that these proteins might perform a biochemical function that was evolutionarily conserved (Figure S2). Furthermore, our study indicated that purified wild-type RBH1 protein had a significantly higher capacity to degrade PGA than the rbh1 protein (Figure 5C). The degradation of pectin in the *rbh1* mutant plants was inhibited (Figure 5D–G). These results indicate that *RBH1* regulates panicle development through the pectin degradation pathway. The *RBH1* is the first pectin lyase gene reported to be involved in the regulation of panicle morphogenesis in rice. In the present study, we independently identify the apical spikelet defect phenotype of *rbh1* (Figure 1). The results of gene cloning suggest that *RBH1* is allelic to *Dwarf and early-senescence leaf 1* (*DEL1*) (Figure 3). The previously identified *DEL1* gene is involved in the induction of leaf senescence. Although *DEL1* is highly expressed in panicle, the *del1* exhibits early leaf senescence rather than an obvious apical panicle defect [58]. More importantly, the *del1* mutant in the Nipponbare genetic background was caused by a single nucleotide substitution, whereas the *rbh1* in the genetic background of Zhonghua 11 was caused by the deletion of the 4^th^ exon. Hence, we propose that the phenotypic disparity between *rbh1* and *del1* is due to the protein dosage effect or rice variety differences.

### 3.3. The Potential role of ROS in Plant Growth and Panicle Development

The ROS signal is highly conserved among aerobic organisms and is required for development, differentiation, redox level, stress signaling, interactions with other organisms, and cell death [59,60]. Early studies focused on the potential toxic effects of ROS, whereby high concentration of ROS is deleterious because it triggers oxidative damages [61]. Moreover, the *rbh1-1* mutant with significantly increased ROS level (Figure 6) leads us to suggest that high ROS level is detrimental to panicle development in rice. Recently, the rice *abnormal inflorescence meristem 1* (*aim1*) mutant exhibited mutant phenotype with reduced root length and decreased root meristem activity. Treatment with exogenous hydrogen peroxide increased ROS accumulation and substantially restored root length. It was demonstrated that the increased ROS concentration promoted root meristem activity [62]. In addition, decreased ROS concentration suppressed cellular proliferation [63,64]. Understanding the dual role (beneficial/detrimental) of ROS is important for studying the function of ROS in plant growth and development. Mittler holds the view that excessively high and low ROS concentrations are both detrimental to plant [65]. Hence, maintaining ROS level in an appropriate range could promote the normal growth and development. In consideration of significantly increased ROS level in *rbh1-1* mutant, we speculate that *RBH1* may ensure the normal development of the apical panicle through maintaining the ROS level in an appropriate range.

## 4. Materials and Methods 

### 4.1. Plant Materials and Growth Conditions

The two T-DNA insertion lines, *rbh1-1* and *rbh1-2* of rice were identified from the T-DNA insertion mutant library [66]. The *rbh1-1* was used for functional analysis of *RBH1*. Rice plants were cultivated in the experimental field at the Huazhong Agriculture University in Wuhan, China.

### 4.2. Gene Cloning

The flanking genomic sequence of the T-DNA insertion site was amplified by thermal asymmetric interlaced-polymerase chain reaction (TAIL-PCR) [40]. A BLAST search of the flanking sequence against the Rice Genome Annotation database was performed (http://rice.plantbiology.msu.edu). Genotyping of the *rbh1-1* segregating population by PCR was performed using primers P1, P2 and P3. P1 and P2 were gene-specific primers targeted to two sides of the T-DNA insertion site, and P3 bound to the border of the T-DNA. The T-DNA element (approximate 10 kb) between the P1, P2 primer sites was too large to be amplified under the specific conditions we used. The PCR condition was as follows: 94 °C for 5 min; 28 cycles of 94 °C for 45 s, 57 °C for 45 s, and 72 °C for 1 min; and then a final extension at 72 °C for 7 min. Genotyping of the *rbh1-2* segregating population by PCR was performed using primers P4, P5, and P6 according to the method as mentioned above. All primers used in this study are listed in Supplementary Table S3.

### 4.3. RNA Extraction, RT-PCR and qRT-PCR

Total RNA was extracted from various tissues using the Trizol reagent (Invitrogen) according to the manufacturer’s instructions. The first-strand cDNA was synthesized using 4 μg of RNA and the M-MLV Reverse Transcriptase (Invitrogen) according to the manufacturer’s instructions. The qRT–PCR carried out in a total volume of 25 μL containing the reverse-transcribed product (6 μL), gene-specific primers (0.25 mM), and SYBR Green Master Mix (12.5 μL, Roche) was performed according to the manufacturer’s instructions. The qRT–PCR conditions were as follows: 95 °C for 2 min, followed by 45 cycles of 95 °C for 10 s, and 60 °C for 30 s. The qRT–PCR was operated with optical 96 or 384-well plate in an ABI PRISM 7500 PCR instrument (Applied Biosystems). The rice *UBI* gene was used for normalization. The 2^−^^△△CT^ method was used to calculate relative expression level [67]. The sequences of the primers used for qRT-PCR are listed in Supplementary Table S3.

### 4.4. Complementation Test

An 11-kb genomic DNA fragment, containing the entire ORF, 2.5 kb upstream and 5.3 kb downstream of *RBH1*, was constructed into the binary vector pCAMBIA2301. The recombinant binary vector was named pC-RBH1. The empty pCAMBIA2301 was also used as a negative control. Both plasmids were electroporated into the *Agrobacterium tumefaciens* strain EHA105, and were transformed into *rbh1-1* mutant callus as described previously [66].

### 4.5. In Situ Hybridization

Panicle samples from different developmental stages were fixed in formaldehyde–acetic acid–ethanol (FAA, 50% ethanol, 5% acetic glacial and 3.7% formaldehyde) for 16h at 4 °C and were then replaced with 70% ethanol twice and dehydrated with 95% ethanol, substituted with xylene, embedded in paraffin, and sectioned to 8–10 µm. *RBH1* CDS fragments were amplified with the primer pairs RBH1-RT-S/AS and then ligated into the pGEM-T vector (Promega). The probe was then transcribed in vitro from the T7 or SP6 promoter with polymerase using a digoxigenin RNA labeling kit (Roche). RNA-RNA in situ hybridization and immunologic detection of the hybridized probes were performed according to the protocol described previously [68]. The antisense probe was used to detect the signal and the sense probe was used as negative control. The sequences of the primers used are listed in Supplementary Table S3.

### 4.6. Immunohistochemical Assay

Panicle samples were fixed in formaldehyde–acetic acid–ethanol (FAA, 50% ethanol, 5% acetic glacial and 3.7% formaldehyde) and then embedded in paraffin for sectioning. Briefly, sections on glass slides were blocked with 3% BSA in PBS (pH 7.2) for 30 min. Then sections were washed with PBS and incubated with monoclonal antibody JIM5, LM18 (Plant Probes, 1:10 dilution) (www.plantprobes.net) for 2 h at 37 °C. After washing with PBS, secondary anti-rat antibody conjugated to fluorescein-isothiocyanate (anti-rat/FITC, IgM, Bioss, 1:100 dilution) was applied for 1h at 37 °C in the dark. Finally, sections were washed with PBS and mounted in PBS/glycerol-based anti-fade solution (5% n-propyl gallate in 90% glycerol/10% PBS) for observation using an Olympus BX61 fluorescence microscope (Olympus, Japan).

### 4.7. Expression, Purification of RBH1 Protein, SDS-PAGE and Enzyme Activity of RBH1 Protein

The truncated coding sequence of RBH1 (51-465aa) and rbh1 (51-422aa) was amplified with primers pMAL-C2X-RBH1-51aa-S, pMAL-C2X-RBH1-465aa-AS and pMAL-C2X-RBH1-422aa-AS and cloned into the pMAL-C2X vector (New England Biolabs), then introduced into Transetta (DE3) cell (TransGen Biotech). The target protein was purified with Amylose Resin (New England Biolabs) according to the manufacturer’s instructions. The sequences of the primers used are listed in Supplementary Table S3. The purity and concentration of the recombinant protein were tested by SDS-PAGE (Figure 5A,B).

The procedure of SDS-PAGE was referred to Laemmli-SDS-PAGE [69]. Please note that increasing the pH to 9.2 in separating gel can greatly improve the efficiency of the experiment and maintain the stability of the protein [70]. The protein sample was mixed with loading buffer and boiled at 95 °C heating block for 10 min, then fast centrifuged for 1 min and placed at room temperature for electrophoresis. The protein sample and 2~3 μL of protein MW marker (Thermo Fisher Scientific) were loaded into the wells. The electrophoresis was operated according to the manufacturer’s instructions (BIO-RAD). The gel was stained with coomassie blue. 

Pectate lyase activity was assayed in the reaction buffer (50 mM Tris-HCl, 1 mM CaCl2, pH 8.8). The reaction buffer contained 2.5 mg/mL of polygalacturonic acid (Sigma). Each 1mL reaction was initiated with 30 μg of purified protein and incubated for 30 min at 40 °C. Then the absorbance data was collected at 235 nm. Units of activity are expressed as nanomoles product per min per mg added protein. 

### 4.8. ROS Detection

The procedure of DAB staining was referred to previous report [71]. 5 mg/mL DAB solution (prepared in double distilled water) (DAB powder, Sangon Biotech) was added to the young panicle. The young panicles were immersed in DAB solution by gently vacuum infiltrating the samples for 5 min in a dessicator and then the samples were covered with aluminium foil. Following the samples were incubated on a standard laboratory shaker for 4–5 h at 80–100 rpm at room temperature. After incubation, the dyed samples were eluted successively with chloralhydrate solution (chloralhydrate 50 g; ddH_2_O 15 mL; glycerol 10mL) and absolute alcohol. The images were captured using a stereomicroscope by keeping the samples on a slide. 

A fresh panicle sample (0.5 g) was placed in a precooled mortar and ground into homogenate on an ice bath. The homogenate was transferred into a centrifuge tube and centrifuged at 12,000× *g* for 10 min (4 °C). The supernatant was aspirated and put on the ice for testing. POD activity test was referred to instructions for use of POD activity assay kit (Solarbio tech), then the absorbance data was collected at 470 nm. Units of POD activity were defined as changed absorbance per ml reaction solution per mg added samples.

### 4.9. Data Analysis

All data were analyzed in the GraphPad Prism 6 software. The *p* values of our data were calculated with a two-tailed Student’s t-test. The *p* < 0.01 indicates that the experiment data was statistical significance.

## Data Availability

The data presented in this study are available on request from the corresponding author.

## Supplementary Materials

The following are available online at https://www.mdpi.com/2223-7747/10/2/271/s1, Figure S1: Identification of *rbh1-2*, Figure S2: RBH1 belongs to the pectate lyase family, Figure S3: TEM analyses of spikelet in the WT (A) and *rbh1-1* (B), Table S1: The panicle growth data of *rbh1-1* mutant and WT, Table S2: Observation of the phenotype of the single copy complement plants, Table S3: Primers for plasmid constructions, expression analysis, Genotype test in our study.

## References

[1] Zhu Q.H., Hoque M., Dennis E.S., Upadhyaya N.M. (2003). Ds tagging of BRANCHED FLORETLESS 1 (BFL1) that mediates the transition from spikelet to floret meristem in rice (Oryza sativa L). BMC Plant. Biol..

[2] Bai X., Huang Y., Mao D., Wen M., Zhang L., Xing Y. (2016). Regulatory role of FZP in the determination of panicle branching and spikelet formation in rice. Sci. Rep..

[3] Komatsu M., Chujo A., Nagato Y., Shimamoto K., Kyozuka J. (2003). FRIZZY PANICLE is required to prevent the formation of axillary meristems and to establish floral meristem identity in rice spikelets. Development.

[4] Kato T., Horibata A. (2011). A novel frameshift mutant allele, fzp-10, affecting the panicle architecture of rice. Euphytica.

[5] Komatsu K., Maekawa M., Ujiie S., Satake Y., Furutani I., Okamoto H., Shimamoto K., Kyozuka J. (2003). LAX and SPA: Major regulators of shoot branching in rice. Proc. Natl. Acad. Sci. USA.

[6] Li X., Qian Q., Fu Z., Wang Y., Xiong G., Zeng D., Wang X., Liu X., Teng S., Hiroshi F. (2003). Control of tillering in rice. Nat. Cell Biol..

[7] Oikawa T., Kyozuka J. (2009). Two-Step Regulation of LAX PANICLE1 Protein Accumulation in Axillary Meristem Formation in Rice. Plant. Cell.

[8] Tabuchi H., Zhang Y., Hattori S., Omae M., Shimizu-Sato S., Oikawa T., Qian Q., Nishimura M., Kitano H., Xie H. (2011). LAX PANICLE2 of Rice Encodes a Novel Nuclear Protein and Regulates the Formation of Axillary Meristems. Plant. Cell.

[9] Bai J., Zhu X., Wang Q., Zhang J., Chen H., Dong G., Zhu L., Zheng H., Xie Q., Nian J. (2015). Rice TUTOU1 Encodes a Suppressor of cAMP Receptor-Like Protein That Is Important for Actin Organization and Panicle Development. Plant. Physiol..

[10] Heng Y., Wu C., Long Y., Luo S., Ma J., Chen J., Liu J., Zhang H., Ren Y., Wang M. (2018). OsALMT7 Maintains Panicle Size and Grain Yield in Rice by Mediating Malate Transport. Plant. Cell.

[11] Hayashi S., Wakasa Y., Takahashi H., Kawakatsu T., Takaiwa F. (2011). Signal transduction by IRE1-mediated splicing of bZIP50 and other stress sensors in the endoplasmic reticulum stress response of rice. Plant. J..

[12] Lu S.J., Yang Z.T., Sun L., Sun L., Song Z.T., Liu J.X. (2012). Conservation of IRE1-Regulated bZIP74 mRNA Unconventional Splicing in Rice (Oryza sativa L.) Involved in ER Stress Responses. Mol. Plant..

[13] Wang Q.L., Sun A.Z., Chen S.T., Chen L.S., Guo F.Q. (2018). SPL6 represses signalling outputs of ER stress in control of panicle cell death in rice. Nat. Plants.

[14] Peng Y., Hou F., Bai Q., Xu P., Liao Y., Zhang H., Gu C., Deng X., Wu T., Chen X. (2018). Rice Calcineurin B-Like Protein-Interacting Protein Kinase 31 (OsCIPK31) Is Involved in the Development of Panicle Apical Spikelets. Front. Plant. Sci..

[15] Vincken J.P., Schols H.A., Oomen R.J., McCann M.C., Ulvskov P., Voragen A.G., Visser R.G. (2003). If Homogalacturonan Were a Side Chain of Rhamnogalacturonan I. Implications for Cell Wall Architecture. Plant. Physiol..

[16] Voragen A.G.J., Coenen G.J., Verhoef R.P., Schols H.A. (2009). Pectin, a versatile polysaccharide present in plant cell walls. Struct. Chem..

[17] O’Neill M., Albersheim P., Darvill A. (1990). The Pectic Polysaccharides of Primary Cell Walls. Meth. Plant. Biochem..

[18] Cosgrove D.J. (2000). Expansive growth of plant cell walls. Plant. Physiol. Biochem..

[19] Knox J.P. (1992). Cell adhesion, cell separation and plant morphogenesis. Plant. J..

[20] Fischer R.L., Bennett A.B. (1991). Role of Cell Wall Hydrolases in Fruit Ripening. Annu. Rev. Plant. Biol..

[21] Wen F., Zhu Y., Hawes M.C. (1999). Effect of Pectin Methylesterase Gene Expression on Pea Root Development. Plant. Cell.

[22] Roberts J.A., Whitelaw C.A., Gonzalez-Carranza Z.H., McManus M.T. (2000). Cell Separation Processes in Plants—Models, Mechanisms and Manipulation. Ann. Bot..

[23] Hongo S., Sato K., Yokoyama R., Nishitani K. (2012). Demethylesterification of the Primary Wall by PECTIN METHYLESTERASE35 Provides Mechanical Support to the Arabidopsis Stem. Plant. Cell.

[24] Celia M.R.M., John O., Seymour G.B. (2002). Pectate lyases, cell wall degradation and fruit softening. J. Exp. Bot..

[25] Peaucelle A., Louvet R., Johansen J.N., Höfte H., Laufs P., Pelloux J., Mouille G. (2008). Arabidopsis Phyllotaxis Is Controlled by the Methyl-Esterification Status of Cell-Wall Pectins. Curr. Biol..

[26] Sun L., Nocker S.V. (2010). Analysis of promoter activity of members of the PECTATE LYASE-LIKE (PLL) gene family in cell separation in Arabidopsis. BMC Plant Biol..

[27] Mayans O., Scott M., Connerton I., Gravesen T., Benen J., Visser J., Pickersgill R., Jenkins J.A. (1997). Two crystal structures of pectin lyase A from Aspergillus reveal a pH driven conformational change and striking divergence in the substrate-binding clefts of pectin and pectate lyases. Structure.

[28] Herron S.R., Benen J.A.E., Scavetta R.D., Visser J., Jurnak F. (2000). Structure and function of pectic enzymes: Virulence factors of plant pathogens. Proc. Natl. Acad. Sci. USA.

[29] Starr M.P., Moran F. (1962). Eliminative Split of Pectic Substances by Phytopathogenic Soft-Rot Bacteria. Science.

[30] Palusa S.G., Golovkin M., Shin S.-B., Richardson D.N., Reddy A.S. (2007). Organ-specific, developmental, hormonal and stress regulation of expression of putative pectate lyase genes in Arabidopsis. New Phytol..

[31] Wu H.B., Wang B., Chen Y., Liu Y.G., Chen L. (2013). Characterization and fine mapping of the rice premature senescence mutant ospse1. Theor. Appl. Genet..

[32] Wing R.A., Yamaguchi J., Larabell S.K., Ursin V.M., McCormick S. (1990). Molecular and genetic characterization of two pollen-expressed genes that have sequence similarity to pectate lyases of the plant pathogen Erwinia. Plant. Mol. Biol..

[33] Wu Y., Qiu X., Du S., Erickson L. (1996). PO149, a new member of pollen pectate lyase-like gene family from alfalfa. Plant. Mol. Biol..

[34] Kulikauskas R., McCormick S. (1997). Identification of the tobacco and Arabidopsis homologues of the pollen-expressed LAT59 gene of tomato. Plant. Mol. Biol..

[35] Jiang J., Yao L., Miao Y., Cao J. (2013). Genome-wide characterization of the pectate lyase-like (PLL) genes in Brassica rapa. Mol. Genet. Genom..

[36] Turcich M.P., Hamilton D.A., Mascarenhas J.P. (1993). Isolation and characterization of pollen-specific maize genes with sequence homology to ragweed allergens and pectate lyases. Plant. Mol. Biol..

[37] Vogel J.P., Raab T.K., Schiff C., Somerville S.C. (2002). PMR6, a Pectate Lyase–Like Gene Required for Powdery Mildew Susceptibility in Arabidopsis. Plant. Cell.

[38] Xie F., Murray J.D., Kim J., Heckmann A.B., Edwards A., Oldroyd G.E.D., Downie J.A. (2011). Legume pectate lyase required for root infection by rhizobia. Proc. Natl. Acad. Sci. USA.

[39] Yang Z., Feng S., Tang D., Zhang L., Li Y., Kear P., Huang S., Zhang C. (2020). The mutation of a PECTATE LYASE-LIKE gene is responsible for the Yellow Margin phenotype in potato. Theor. Appl. Genet..

[40] Liu Y.G., Mitsukawa N., Oosumi T., Whittier R.F. (1995). Efficient isolation and mapping of Arabidopsis thaliana T-DNA insert junctions by thermal asymmetric interlaced PCR. Plant. J..

[41] Verhertbruggen Y., Marcus S.E., Haeger A., Ordaz-Ortiz J.J., Knox J.P. (2009). An extended set of monoclonal antibodies to pectic homogalacturonan. Carbohydr. Res..

[42] Held M.A., Be E., Zemelis S., Withers S., Wilkerson C., Brandizzi F. (2011). CGR3: A Golgi-Localized Protein Influencing Homogalacturonan Methylesterification. Mol. Plant..

[43] Yahraus T., Chandra S., Legendre L., Low P.S. (1995). Evidence for a Mechanically Induced Oxidative Burst. Plant. Physiol..

[44] Monshausen G.B., Bibikova T.N., Weisenseel M.H., Gilroy S. (2009). Ca2+ Regulates Reactive Oxygen Species Production and pH during Mechanosensing in Arabidopsis Roots. Plant. Cell.

[45] Kärkönen A., Kuchitsu K. (2015). Reactive oxygen species in cell wall metabolism and development in plants. Phytochemistry.

[46] Apel K., Hirt H. (2004). Reactive oxygen species: Metabolism, oxidative stress, and signal transduction. Annu. Rev. Plant. Biol..

[47] Nierhaus K.H. (1982). Structure, Assembly, and Function of Ribosomes. Curr. Topics Microbiol. Immunol..

[48] Ito Y., Saisho D., Nakazono M., Tsutsumi N., Hirai A. (1997). Transcript levels of tandem-arranged alternative oxidase genes in rice are increased by low temperature. Gene.

[49] Saika H., Ohtsu K., Hamanaka S., Nakazono M., Tsutsumi N., Hirai A. (2002). AOX1c, a novel rice gene for alternative oxidase; comparison with rice AOX1a and AOX1b. Genes Genet. Syst..

[50] Magneschi L., Perata P. (2008). Rice germination and seedling growth in the absence of oxygen. Ann. Bot..

[51] Kaminaka H., Morita S., Tokumoto M., Yokoyama H., Masumura T., Tanaka K. (1999). Molecular Cloning and Characterization of a cDNA for an Iron-Superoxide Dismutase in Rice (Oryza sativaL.). Biosci. Biotechnol. Biochem..

[52] Xing Y., Zhang Q. (2010). Genetic and Molecular Bases of Rice Yield. Annu. Rev. Plant. Biol..

[53] Domingo C., Roberts K., Stacey N.J., Connerton I., Ruíz-Teran F., McCann M.C. (2002). A pectate lyase from Zinnia elegans is auxin inducible. Plant. J..

[54] Milioni D., Sado P.-E., Stacey N.J., Domingo C., Roberts K.J., McCann M.C. (2001). Differential expression of cell-wall-related genes during the formation of tracheary elements in the Zinnia mesophyll cell system. Plant. Mol. Biol..

[55] Medina-Escobar N., Cárdenas J., Moyano E., Caballero J.L., Garcίa-Limones C. (1997). Cloning, molecular characterization and expression pattern of a strawberry ripening-specific cDNA with sequence homology to pectate lyase from higher plants. Plant. Mol. Biol..

[56] Medina-Suarez R., Manning K., Fletcher J., Aked J., Bird C.R., Seymour G.B. (1997). Gene Expression in the Pulp of Ripening Bananas (Two-Dimensional Sodium Dodecyl Sulfate-Polyacrylamide Gel Electrophoresis of in Vitro Translation Products and cDNA Cloning of 25 Different Ripening-Related mRNAs). Plant. Physiol..

[57] Zheng Y., Yan J., Wang S., Xu M., Huang K., Chen G., Ding Y. (2018). Genome-wide identification of the pectate lyase-like (PLL) gene family and functional analysis of two PLL genes in rice. Mol. Genet. Genom..

[58] Leng Y., Yang Y., Ren D., Huang L., Dai L., Wang Y., Chen L., Tu Z., Gao Y., Li X. (2017). A Rice PECTATE LYASE-LIKE Gene Is Required for Plant Growth and Leaf Senescence. Plant. Physiol..

[59] Baxter A., Mittler R., Suzuki N. (2014). ROS as key players in plant stress signalling. J. Exp. Bot..

[60] Ortega-Galisteo A.P., Rodríguez-Serrano M., Pazmiño D.M., Gupta D.K., Sandalio L.M., Romero-Puertas M.C. (2012). S-Nitrosylated proteins in pea (Pisum sativum L.) leaf peroxisomes: Changes under abiotic stress. J. Exp. Bot..

[61] Mittler R., Vanderauwera S., Suzuki N., Miller G., Tognetti V.B., Vandepoele K., Gollery M., Shulaev V., Van Breusegem F. (2011). ROS signaling: The new wave?. Trends Plant. Sci..

[62] Xu L., Zhao H., Ruan W., Deng M., Wang F., Peng J., Luo J., Chen Z., Yi K. (2017). ABNORMAL INFLORESCENCE MERISTEM1 Functions in Salicylic Acid Biosynthesis to Maintain Proper Reactive Oxygen Species Levels for Root Meristem Activity in Rice. Plant. Cell.

[63] West A.P., Brodsky I.E., Rahner C., Woo D.K., Erdjument-Bromage H., Tempst P., Walsh M.C., Choi Y., Shadel G.S., Ghosh S. (2011). TLR signalling augments macrophage bactericidal activity through mitochondrial ROS. Nat. Cell Biol..

[64] Mittal M., Siddiqui M.R., Tran K., Reddy S.P., Malik A.B. (2014). Reactive Oxygen Species in Inflammation and Tissue Injury. Antioxidants Redox Signal..

[65] Mittler R. (2017). ROS Are Good. Trends Plant. Sci..

[66] Wu C., Li X., Yuan W., Chen G., Kilian A., Li J., Xu C., Li X., Zhou D.X., Wang S. (2003). Development of enhancer trap lines for functional analysis of the rice genome. Plant. J..

[67] Livak K.J., Schmittgen T.D. (2001). Analysis of relative gene expression data using real-time quantitative PCR and the 2^−ΔΔCT^ Method. Methods.

[68] DeBlock M., Debrouwer D. (1993). RNA-RNA in Situ Hybridization Using Digoxigenin-Labeled Probes: The Use of High-Molecular-Weight Polyvinyl Alcohol in the Alkaline Phosphatase Indoxyl-Nitroblue Tetrazolium Reaction. Anal. Biochem..

[69] He F. (2011). Laemmli-SDS-PAGE. BIO-PROTOCOL.

[70] Makowski G., Ramsby M. (1993). pH Modification to Enhance the Molecular Sieving Properties of Sodium Dodecyl Sulfate-10% Polyacrylamide Gels. Anal. Biochem..

[71] Daudi A., O’Brien J.A. (2012). Detection of Hydrogen Peroxide by DAB Staining in Arabidopsis Leaves. BIO-PROTOCOL.

