# Supplementary material for: Rice RBH1 Encoding A Pectate Lyase is Critical for Apical Panicle Development
Source: Plants (Basel). 2021 Jan 30;10(2):271. doi: 10.3390/plants10020271 (PMC7912155; doi:10.3390/plants10020271)
Supplement: Supplementary file 1 [file plants-10-00271-s001.pdf]

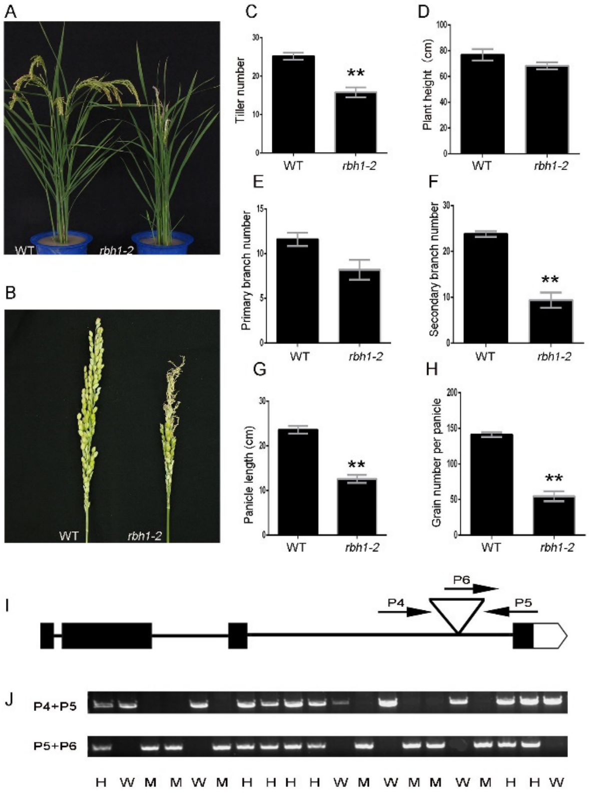

Figure S1. Identification of *rbh1-2*. (**A**) Phenotype comparison of wild type (WT) (left) and *rbh1-2* (right). (**B**) Phenotype comparison of WT panicle (left) and *rbh1-2* representative panicle (right). (**C-H**) Agronomic trait analysis of tiller number, plant height, primary branch number, secondary branch number, panicle height and grain number per panicle between WT and *rbh1-2*. Data are presented as means ± SE (n =5). **P< 0.01 (Student's t test). (**I**) The structure of *RBH1* and Transfer DNA (T-DNA) insertion sites. Black lines represent the intron, the black boxes represent the exon, the white arrow represents the 3'UTR region, and the white triangle represents the T-DNA insertion site. P4 and P5 are genomic primers on both sides of T-DNA insertion site, P6 is a T-DNA boundary primer. (**J**) PCR genotyping in the *rbh1-2* segregant. All plants homozygous for T-DNA insertion showed the positive band with P5+P6 primers and negative with P4+P5 primers have mutant phenotype (M). All plants of WT (W) for T-DNA insertion showed the positive band with P4+P5 primers and the negative with P5+P6 primers, and plants heterozygous (H) for T-DNA insertion showed the both positive bands have normal phenotype.

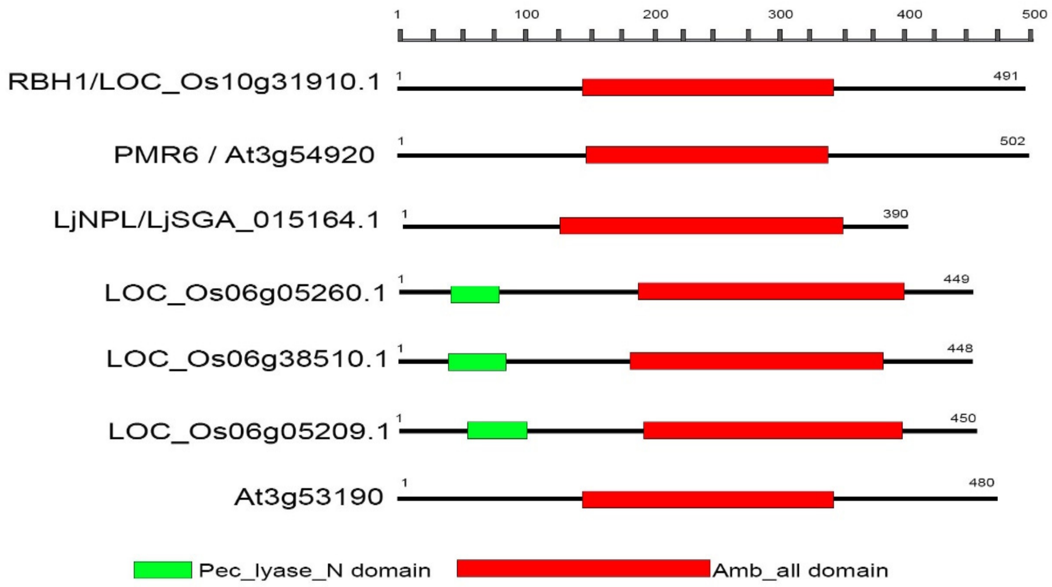

Figure S2. RBH1 belongs to the pectate lyase family.

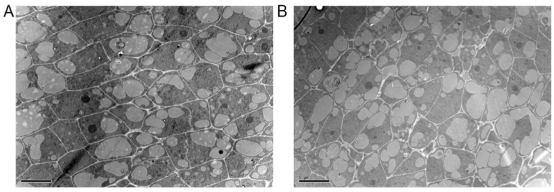

Figure S3. Transmission Electron Microscope (TEM) analyses of spikelet in the WT (**A**) and *rbh1-1* (**B**). Scale bar = 5 μm.

Table S1. The panicle growth data of *rbh1-1* mutant and wild type. Data are presented as means ± SE (n = 5).

Day After Germination (day)	Panicle Length of Wild Type (cm)	Panicle Length of *rbh1-1* (cm)
40	0	0
54	1.12 ± 0.10	0.152 ± 0.01
70	20.14 ± 0.73	12.68 ± 1.01
87	22.66 ± 1.25	16.98 ± 0.55

Table S2. Observation of the phenotype of the complement transgenic plants.

Serial Number	Num. of plant	Num. of Mutant	Wild type	χ^2
pC2301-RBH1-T_1-1	20	5	15	0.06667
pC2301-RBH1-T_1-2	26	7	19	0.20513
pC2301-RBH1-T_1-3	35	4	31	2.75238
pC2301-RBH1-T_1-4	40	6	34	1.63333

Table S3. Primers for plasmid constructions, expression analysis, Genotype test in our study.

Primer name	Sequence(5'-3')
Plasmid constructions	
pMAL-C2X-RBH1-51aa-S	aaaaGAATTCTCGCGGCGGCGGATGCAGA
pMAL-C2X-RBH1-465aa-AS	aaaaAAGCTTTCAGCCGTTGTCCCTGGGGCCG
pMAL-C2X-RBH1-422aa-AS	aaaaAAGCTTTCACCAATTGCCGCCGCTGCCA
Expression analysis	

RBH1-RT-S	GAGGCCATCTACGACAAGG
RBH1-RT-AS	CACAATGGGTGCAAGCATA
Ubiquitin-QRT-S	CGACCCGTTCATCACCACCGAC
Ubiquitin-QRT-AS	ACGATTGATTTAACCAGTCCATGA
AOX1a-QRT-S	CTTCGCATCGGACATCCATTA
AOX1a-QRT-AS	TCCTCGGCAGTAGACAAACATC
AOX1b-QRT-S	CCTGCTCAGTTCATCACCATCA
AOX1b-QRT-AS	GCATAAAACGGAGTGACAATAGC
SODB-QRT-S	TCCGCCGTATAAACTTGATGCCCT
SODB-QRT-AS	TGGGTTGCCGTTGTTGTATGCTTC
SODA1-QRT-S	ATCTGGATGGGTGTGGCTAGCTTT
SODA1-QRT-AS	AGTACGCATGCTCCCAGACATCAA
catA-QRT-S	CAACCGCAACGTCGACAACTTCTT
catA-QRT-AS	TTCACCGGCAGCATCAGGTAGTTT
catB-QRT-S	GCTTGCTTTCTGCCCAGCGATAAT
catB-QRT-AS	AAATAGTTTGGGCCAAGACGGTGC
Genotype test	
P1	CCCGTTTGTTTCTGACTAAGATC
P2	AAGATTCGGTGAGGCTGACAT
P3	AATCCAGATCCCCCGAATTA
P4	CAGTGGCGAGCAAGTGGAG
P5	GGGTTGATTAAACACCGGGATA
P6	TGCAGGTTCTCTCCAAATGA
